# p53 shapes genome-wide and cell type-specific changes in microRNA expression during the human DNA damage response

**DOI:** 10.4161/15384101.2015.942209

**Published:** 2014-10-30

**Authors:** Hiroyoshi Hattori, Rekin’s Janky, Wilfried Nietfeld, Stein Aerts, M Madan Babu, Ashok R Venkitaraman

**Affiliations:** 1 University of Cambridge; Medical Research Council Cancer Unit; Hutchison/MRC Research Center; Cambridge, UK; 2 Medical Research Council Laboratory of Molecular Biology; Cambridge, UK; 3 Center for Human Genetics; KU Leuven; Campus Gasthuisberg; Leuven, Belgium; 4 Max Planck Institute for Molecular Genetics; Berlin-Dahlem, Germany; † These authors contributed equally to the work.; ‡ Present address: Laboratory of Advanced Therapy; Clinical Research Center; National Hospital Organization; Nagoya Medical Center; Aichi, Japan

**Keywords:** p53, micro-RNA, DNA damage response, next-generation sequencing, computational analysis, clinical outcome

## Abstract

The human DNA damage response (DDR) triggers profound changes in gene expression, whose nature and regulation remain uncertain. Although certain micro-(mi)RNA species including miR34, miR-18, miR-16 and miR-143 have been implicated in the DDR, there is as yet no comprehensive description of genome-wide changes in the expression of miRNAs triggered by DNA breakage in human cells. We have used next-generation sequencing (NGS), combined with rigorous integrative computational analyses, to describe genome-wide changes in the expression of miRNAs during the human DDR. The changes affect 150 of 1523 miRNAs known in miRBase v18 from 4–24 h after the induction of DNA breakage, in cell-type dependent patterns. The regulatory regions of the most-highly regulated miRNA species are enriched in conserved binding sites for p53. Indeed, genome-wide changes in miRNA expression during the DDR are markedly altered in *TP53-/-* cells compared to otherwise isogenic controls. The expression levels of certain damage-induced, p53-regulated miRNAs in cancer samples correlate with patient survival. Our work reveals genome-wide and cell type-specific alterations in miRNA expression during the human DDR, which are regulated by the tumor suppressor protein p53. These findings provide a genomic resource to identify new molecules and mechanisms involved in the DDR, and to examine their role in tumor suppression and the clinical outcome of cancer patients.

## Abbreviations

DDR: DNA damage response
miRNA: micro-RNA
NGS: next-generation sequencing
TP53: tumour protein p53
NF-k B: nuclear factor-k B
AP-1: activator protein-1
E2F1: transcription factor E2F1
FoxM1: forkhead box protein M1
ionizing radiation: IR
double stranded DNA breaks: DSBs
tRNA: transfer RNA
snoRNA: small nucleolar RNA
scRNA: small cytoplasmic RNA
snRNA: small nuclear RNA
misc RNA: miscellaneous RNA
TF: transcription factor

## Introduction

The response of human cells to DNA damage (DDR) is essential for the maintenance of genome stability. The DDR not only involves well-characterized changes in protein localization or modification, but also profound changes in gene expression that are less well understood. Thus, DDR signaling alters gene transcription via a plethora of transcription factors including NF-кB ^1,2^, p53,^3^ AP-1,^4^ E2F1 ^5^ or FoxM1.^6^ Moreover, there is evidence that certain small non-coding RNAs such as micro (mi)RNAs can also modulate changes in gene expression during the DDR. In particular, members of the miR-34 family are induced by DNA damage to regulate the expression of proteins involved in cell cycle progression or cell death.^7,8,9,10,11^ Other miRNA species including miR-182,^12^ miR-16 and miR-143^13,14^ have also been implicated. However, there is as yet no comprehensive description of genome-wide changes in the expression of miRNAs triggered by DNA breakage in human cells.

We have addressed this issue using next-generation sequencing (NGS). Experience in the use of NGS to document changes in small, non-coding RNA expression during a biological process is still limited.^15,16^ Although the method has the theoretical advantages of high sensitivity, wide dynamic range and the capacity to detect alterations in known as well as hitherto undocumented small RNA species,^17^ robust methodologies for its deployment in this setting are still being established.

Here, we provide a methodological blueprint for the characterization by NGS of miRNA expression in human cells via a systematic pipeline of bioinformatics analyzes. We provide a new resource and database describing these changes during the DDR, which not only can be used to compare changes in the human miRNAome during different biological processes, but also can be mined to reveal new molecules and mechanisms underlying the DDR. Our findings reveal a biological role for the tumor suppressor p53 in shaping genome-wide changes in miRNA expression during the DDR in a cell-type specific manner.

## Results

### Genome-wide changes in the expression of small non-coding RNAs are induced by DNA damage in human cells

**Figure 1.**
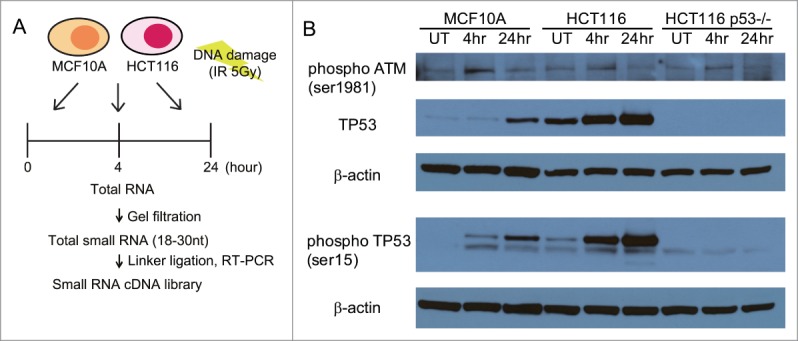
Experimental design for studying genome-wide changes in small non-coding RNA expression during the DDR. **(A)** Outline of the experimental design**.** MCFA10A or HCT116 cells were treated with the 5Gy IR and harvested at each indicated time point. The 18~30 nt fraction of cellular small RNA was isolated by gel filtration. cDNA libraries were generated from this fraction using the Illumina Small RNA Sample Prep Kit before next-generation sequencing. (**B**) Western blots for phosphorylated ATM (Ser1981), TP53 and phosphorylated TP53 (Ser15) in the cell lines used in these studies (MCF10A, HCT116 and HCT116 *TP53-/-*) after DNA damage. Cells exposed to 5Gy IR were harvested at 4 and 24 h after exposure, before extract preparation for blotting. UT refers to untreated cells.

Ionizing radiation (IR) induces several different DNA lesions, including widespread DNA breakage in the form of double-stranded DNA breaks (DSBs), besides single-stranded gaps and nucleotide alterations.^18^ To characterize changes in small non-coding RNA expression during the DDR, we first exposed 2 human cell lines, MCF10A^19^ and HCT116,^20^ to a dose of 5Gy, estimated to create on average 200 DSBs per cell.^21^ Cells were harvested at 0 h (i.e., untreated cells) or at 4 h and 24 h following exposure (**Fig. 1A**). Both MCF10A and HCT116 cell lines exhibited a similar, robust response to IR exposure, evidenced by the phosphorylation of ATM kinase on Ser1981, induction of p53, and its phosphorylation on Ser15 (**Fig. 1B**). Total RNAs extracted from the IR-exposed cells were size-selected for small RNA molecules corresponding to 18-30nt, before cDNA library generation for NGS using the Illumina DGE small RNA prep kit v1.5 (**Fig. 1A**). Capillary-chip electrophoresis was used to confirm the size, quality and concentration of the cDNA libraries before NGS.

The raw reads were processed using a bioinformatics pipeline presented in **Fig. S1A** and mapped to the human genome. The mapped reads were categorized into different RNA species on the basis of their sequence (Methods). In particular, 1211 miRNAs among the mapped reads were identified as known species in miRBase v18^22^ using BLAST,^23^ whereas 308 putative new miRNA species were predicted using MIReNA v2.^24^

**Figure 2.**
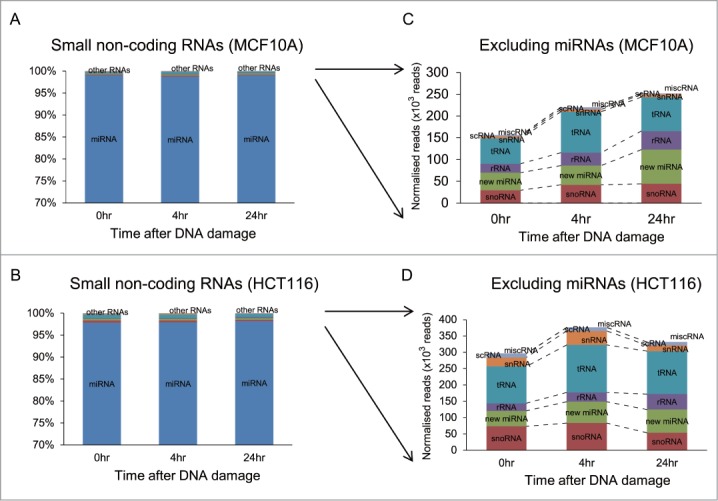
Classes of small non-coding RNAs responsive to DNA damage. Proportions of the different classes of small non-coding RNAs detected after DNA damage among mapped sequence reads in MCF10A (**A**) and in HCT116 (**B**) are summarized in the bar chart as the average fractions of total counts for each class. Y-axis represents the percentages of the number of reads in different classes of small RNAs at different time points. (**C**) and (**D**) enlarge changes in the fractions of small non-coding RNA classes excluding miRNAs. rRNA: ribosomal RNA, scRNA: small cytoplasmic RNA, snRNA: small nuclear RNA, snoRNA: small nucleolar RNA, tRNA: transfer RNA, misc RNA: miscellaneous RNA.

Known miRNAs account for the vast majority of small non-coding RNA species detected in our experimental approach. Approximately 98% of the 17∼22 × 10^6^ mapped reads in each of the 3 samples prepared from MCF10A cells corresponded to known miRNAs (**Fig. 2A**), as did a similar fraction of the 13∼15 × 10^6^mapped reads detected in the corresponding samples from HCT116 cells (**Fig. 2B**). A very small number (0.28% on average) of the mapped reads in both cell types were predicted to encode putative new miRNA species (**Fig. 2C** and **D**). While our method of sample preparation for NGS is designed to extract only small RNA species of length 18-30nt corresponding to miRNAs, we could still detect changes in the expression of certain other small non-coding RNA molecules such as tRNAs (∼0.42%) and small nucleolar (sno)RNAs (∼0.34%) (**Fig. 2C** and **D**), including ACA45, a snoRNA thought to exhibit miRNA-like functions.^25^ However, these changes affect only a small fraction of these small, non-coding RNAs, and given the technical limitations of our sampling procedure, do not capture the majority of such events.

### DDR-induced changes in the expression of known miRNAs exhibit cell-type dependent patterns

**Figure 3.**
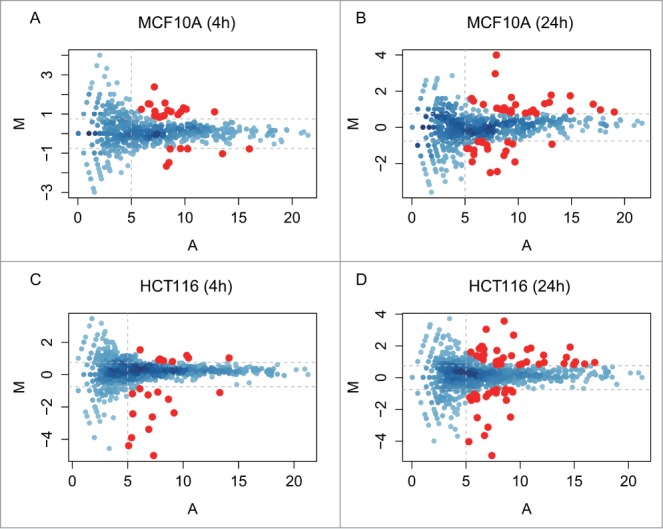
miRNA expression during the DDR in MCF10A and HCT116 cells over time. The MA plots in the panels plot for each miRNA species the values of the log fold change (M) against the average abundance (A). We chose as significant those changes in which M ≥ or ≤0.75 (horizontal dotted lines), and A ≥ 5 (vertical dotted lines), i.e., ≥1.68-fold increase or decrease in differential expression (M) and a count rate of ≥32 (A). The MA plot analysis shows 4 comparisons: **A**, MCF10A 4 h vs 0 h; **B**, MCF10A 24 h vs 0 h; **C**, HCT116 4 h vs 0 h; **D**, HCT116 24 h vs 0 h. miRNAs exhibiting a statistically significant change are color ed in red (p-value < = 0.05).

**Figure 4.**
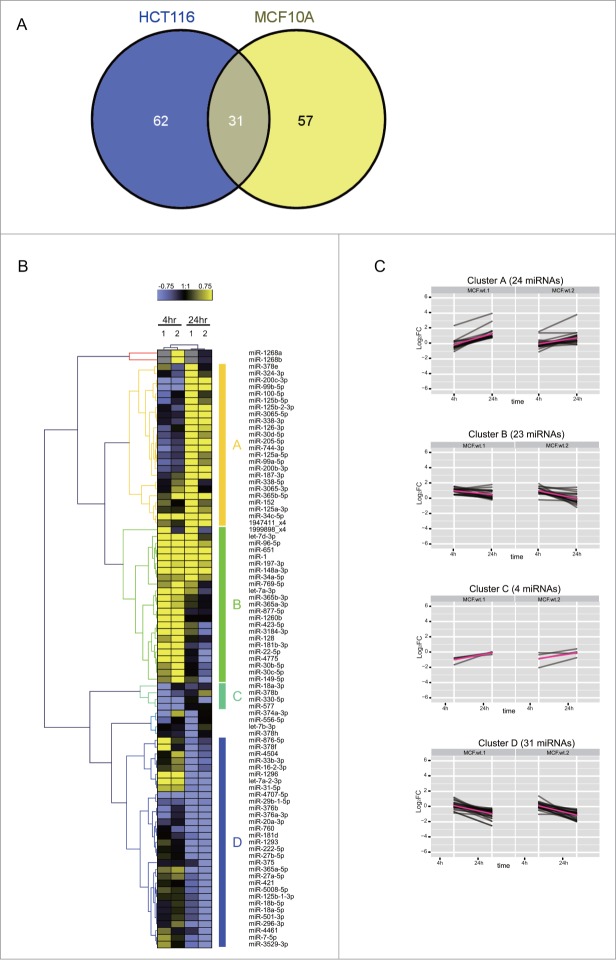
Cell-type dependent patterns of miRNA expression during the DDR. **(A**) shows a Venn diagram comparing the distribution in MCF10A and/or HCT116 cells of 150 DDR-responsive miRNAs whose expression is significantly altered during the DDR by the criteria specified (≥1.68-fold increase or decrease in expression, count rate of ≥32, and significant in at least one of the samples (p ≤ 0.001, Fisher test)). (**B**) shows the heat map of expression patterns for 88 DDR-responsive miRNAs in MCF10A cells at 4 h and 24 h after DNA damage, compared to their expression at 0 h. Two technical replicates (1 and 2) were analyzed for each condition. Each row represents a specific miRNA species. miRNAs were clustered by the pattern of the expression into clusters A–D.

To identify DDR-induced changes in the expression of known miRNAs, we first compared normalized read counts for the identified miRNA species at 4 h and 24 h after IR to untreated control cells at 0 h. The significance of the differential expression for each miRNA was calculated on count data using a binomial approximation to Fisher’s exact test.^26^ The **Fig. 3** shows the log2 fold change values (M) for each species plotted on the y-axis vs. the absolute number of reads (A) on the x-axis. We focused on DDR-induced changes in the expression of miRNAs with corrected p-value < 0.05, excluding any miRNAs of low abundance with A < 5 (i.e., absolute read count <32) and any miRNAs with only a low change in expression with |M| < 0.75 (i.e., 0.59 < FC < 1.68) when compared with the control. Eighty-eight and 93 miRNA species fulfil these criteria at 4 h or 24 h after the exposure to DNA damage of MCF10A and HCT116 cells, respectively. Overall, we found 150 miRNAs whose expression is significantly altered during the DDR, including 31 miRNAs expressed in both MCF10A and HCT116 cell lines (**Fig. 4A**). For convenience, we refer henceforth to these DDR-regulated species as ‘DDR miRNAs’.

Several patterns of altered expression were observed in MCF10A cells (**Fig. 4B**), ranging from simple, sustained induction or repression of expression at 4 h and 24 h, to more complex patterns. We classified the patterns of expression by their kinetics into 4 representative clusters, A-D (**Fig. 4C**). Clusters A and B contain miRNAs whose expression was significantly induced: 23 were maximally induced at 4 h (cluster A) and 24 were maximally induced at 24 h (cluster B). Clusters C and D contain repressed miRNAs, whose expression significantly decreased after DNA damage. Cluster C, which included only 4 miRNAs, exhibited a rapid repression maximal at 4 h, whereas cluster D, including 31 miRNAs, exhibited baseline expression at 4 h with evident repression at 24 h.

**Figure 5.**
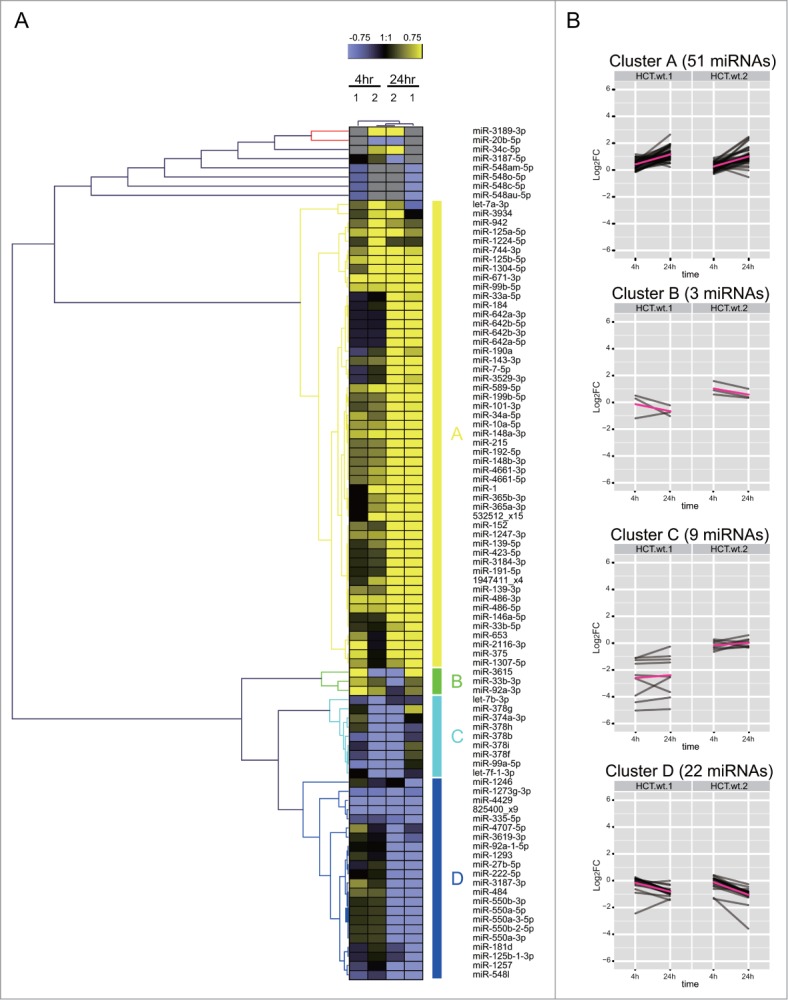
Cell-type dependent patterns of miRNA expression during the DDR. (A) shows the heat map of expression patterns for 93 DDR-responsive miRNAs in HCT116 cells at 4 h and 24 h after DNA damage, compared to their expression at 0 h. Two technical replicates (1 and 2) were analyzed for each condition. Each row represents a specific miRNA species. miRNAs were clustered by (**B**) shows the heat map of expression patterns for 93 DDR-responsive miRNAs in HCT116 cells at 4 h and 24 h after DNA damage, compared to their expression at 0 h.

Similar expression patterns are also observed for the DDR miRNAs in HCT116 (**Fig. 5**). However, the composition of the similarly-regulated clusters is distinct, revealing cell type-specific differences. For example, cluster A in HCT116 corresponds to 51 miRNAs induced mainly at 24 h, but only 6 of them (miRs 125a-5p, 744-3p, 125b-5p, 99b-5p, 152 and 1947411_x4) are shared with MCF10A. Similarly, cluster B in HCT116 corresponds to only 3 miRNAs induced mainly at 4 h but none of them are shared with MCF10A. Intriguingly, 7 DDR miRNAs (miRs 1, 34a-5p, let-7a-3p, 365b-3p, 365a-3p, 423-5p, 3184-3p) are induced at 24 h in HCT116 (cluster A) and at 4 h in MCF10A (cluster B) (**Figs. 4B** and **5A**). Indeed, as a general trend, the DDR miRNAs in HCT116 are not as highly induced at 4 h when compared with MCF10A, but instead, are more highly induced at 24 h (Chi square p-value = 0.009534). MA plot analysis also shows that a larger number of miRNAs were induced at 24 h than at 4 h in HCT116 (**Fig. 3C** and **D**), whereas similar numbers of miRNAs were induced in MCF10A (**Fig. 3A** and **B**). These observations suggest cell-type dependent differences in the kinetics of the induction of DDR miRNAs, with a slower response evident in HCT116 cells.

To further characterize the induced miRNAs, we focused on the most robustly-induced miRNAs after DNA damage using the following filtering criteria: (*a*) the miRNAs must show a consistent induction at 4 h and 24 h, (*b*) the fold change at 4 h or 24 h should be higher than 0.75 (log2 value), (*c*) miRNAs should be significantly induced in both cell lines and/or in 2 experimental replicates. We did not include microRNA isoforms,^27^ which are mature microRNA isoforms that have an altered 3′ end, in our analysis. These criteria detected 23 DDR miRNAs (**Fig. S1B**). Two additional filtering steps were performed to remove redundant miRNAs. First, when 2 mature sequences (eg., -5p and -3p) from the same stem loop were selected (eg., miR-486 and miR-139), we kept the sequence showing the best read coverage (here the -5p fragment) on the premise that it should correspond to the functional species of the stem loop; this step discarded only 2 DNA-damage responsive miRNAs corresponding to star sequences (miR-139-3p, miR-486-3p). Second, when the mature sequences were identical, we selected only one of them (eg., miR-365a-3p and miR-365b-3p). These additional filtering steps left us with a total of 20 DDR miRNAs, 12 of which were induced in MCF10A and 19 in HCT116, with 11 induced in both cell types, 1 in MCF10A alone, and 8 in HCT116 alone (**Table 1**). These findings further speak to the cell-type dependency of changes in miRNA expression noted earlier.

**Table 1.** Human miRNAs whose expression is significantly altered during the DDRgrey

	M value						
	MCF		HCT		*Differential expression		
miRNAs	4 hr	24 hr	4 hr	24 hr	MCF10A	HCT116	Max(M)
miR-34c-5p	2.37	3.98	0.59	3.54	1	1	3.98
1947411_x4	0.37	0.95	0.59	2.65	1	1	2.65
miR-486-5p	Inf	Inf	0.58	1.95	1	1	1.95
miR-148a-3p	1.10	1.77	0.71	1.26	1	1	1.77
miR-152	0.33	0.85	0.23	1.76	1	1	1.76
miR-1247-3p	NA	NA	0.53	1.69	0	1	1.69
miR-1	1.23	0.98	0.77	1.58	1	1	1.58
let-7a-3p	1.55	0.49	0.89	0.50	1	1	1.55
miR-365a-3p	0.91	0.16	0.47	1.43	1	1	1.43
miR-3184-3p	1.10	0.30	0.21	1.39	1	1	1.39
miR-423-5p	1.10	0.30	0.21	1.39	1	1	1.39
miR-139-5p	1.00	−1.00	0.40	1.23	0	1	1.23
miR-125b-5p	−0.54	0.89	1.02	0.87	0	1	1.02
miR-191-5p	0.04	0.29	0.17	0.97	0	1	0.97
miR-34a-5p	0.47	0.77	0.32	0.97	1	1	0.97
miR-96-5p	0.96	0.93	0.51	0.40	1	0	0.96
miR-192-5p	0.18	0.44	0.40	0.93	0	1	0.93
miR-215	0.19	0.46	0.40	0.93	0	1	0.93
miR-148b-3p	0.28	0.26	0.43	0.84	0	1	0.84
miR-146a-5p	−0.64	−0.60	0.16	0.84	0	1	0.84

* Criteria for differential expression is M > 0.75, A > 5, corr p < 0.05; 1, satisfied; 0, not satisfied.

M values are from the replicate 1.

It has previously been reported that NGS methods have greater sensitivity and dynamic range when compared to quantitative (q)RT-PCR based assays for miRNA expression.^17^ Accordingly, we tested if the induction of 15 of the 20 most highly-induced miRNA species identified by NGS was also measurable by qRT-PCR in MCF10A or HCT116 cells exposed to DNA damage. Nine distinct miRNA species exhibited concordance in MCF10A samples, and 7 did so in HCT116 cells (Supplementary material **Fig. S2**), consistent with the slower kinetics and decreased amplitude of induction in the latter cell type observed using NGS (**Figs. 4B** and **5**). Notably, the lack of concordance is observed for miRNAs with low differential expression, consistent with the lack of sensitivity of the qRT-PCR method in detecting small changes in the expression of relatively abundant species.

We adopted the same approach to define the 10 most-robustly repressed miRNAs after DNA damage: 9/10 were repressed in MCF10A and 7 in HCT116, with 6 repressed in both cell types (**Fig. S1C**). Interestingly, the species found specifically repressed in HCT116 alone is a new miRNA, identified as a member of the miR-1273 family using BLASTN against miRNA stem loops annotated in the current miRBase (similarity with hsa-mir-1273a, E-value = 6e-18). We also found 2 members for each of the miR-376 and miR-378 families. These observations once more distinguish cell-type specific patterns in the identity and kinetics of miRNA expression following DNA damage.

### DDR miRNAs are preferentially organized as intragenic clusters

**Figure 6.**
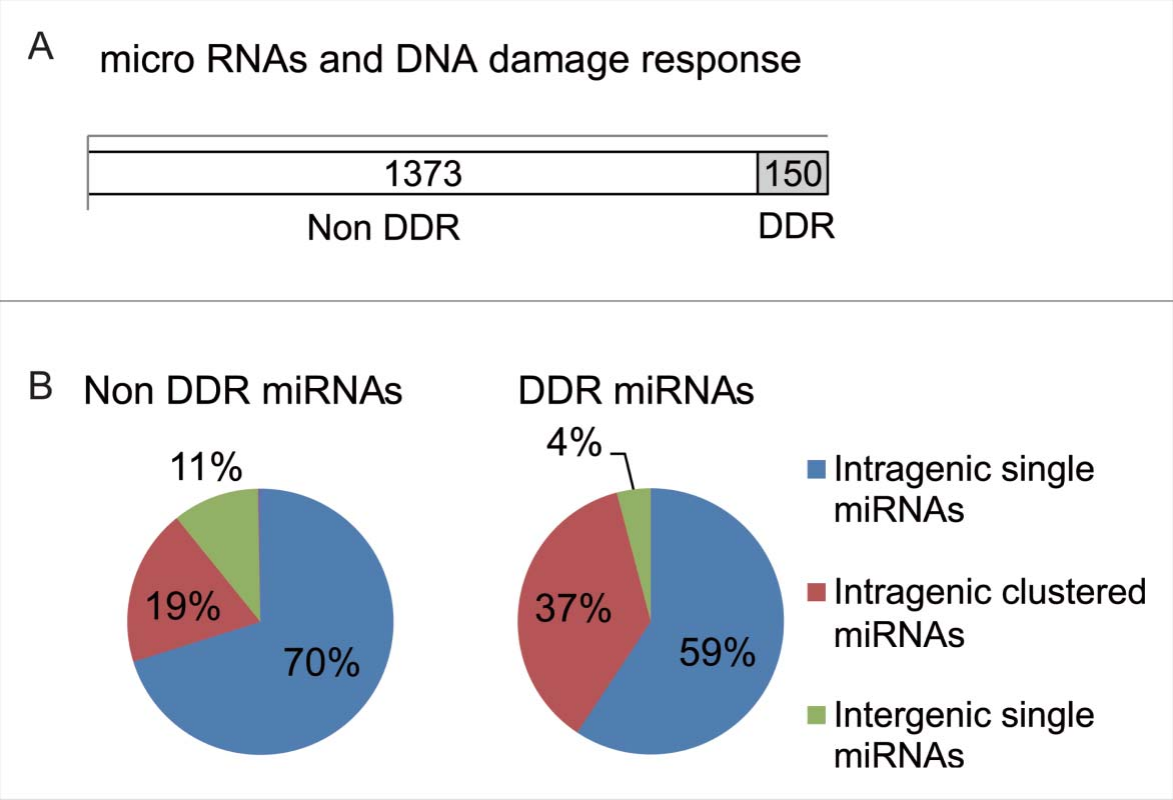
The distribution pattern of DDR miRNAs. (**A**) Shows the distribution in the human genome of loci encoding miRNAs whose expression is altered in response to DNA damage (DDR miRNAs). Open column represents miRNAs that were not induced by DNA damage. Grey color ed column include miRNAs that responded to 5Gy ionizing radiation. (**B**) Pie chart represents the percentages of miRNAs by the site on the genome. Intragenic single miRNA are shown in blue, intragenic miRNAs with the cluster in red and intergenic single miRNAs in green, respectively.

We also tested the distribution of genomic loci of DDR miRNAs against all miRNA-encoding loci in the genome (**Fig. 6A**). For this analysis, each miRNA was assigned a descriptor classifying it as intergenic or intragenic (ie., within the locus of a host gene), and as single or clustered (ie., spacing <10 kb of a second miRNA known in miRBase). **Figure 6B** shows the proportion of the DDR miRNAs and for comparison, species not regulated during the DDR (non-DDR miRNAs) in each of these categories. Distribution of DDR vs. non-DDR miRNAs across the categories was compared using the Fisher Exact Test. About twice as many DDR miRNAs (53/145; 37%) were found within intragenic clustered species compared to non-DDR miRNAs (264/1386; 19%) (pval = 2.73E-5), while the difference observed for intergenic species was not found significant (p val = 1) (**Fig. 6B** and **Table S1**). This suggests that miRNAs organized within clusters in their host genes may be preferentially coordinated by transcriptional regulation during the human DDR. However, we observed no significant difference between miRNAs that are DDR or non-DDR in intragenic as well as intergenic regions (p val = 0.072 for single, p val = 1 for clustered).

### p53 shapes genome-wide changes in the expression of miRNAs after DNA damage in human cells

**Figure 7.**
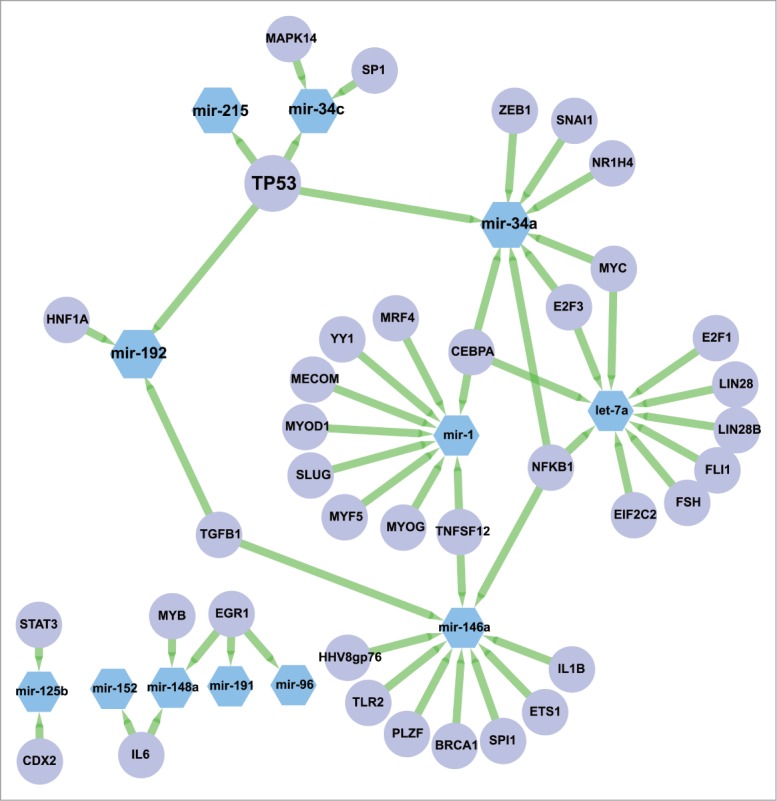
Annotated network between DDR miRNAs and transcription factors. Sub-network corresponding to the DDR miRNAs and their known regulators annotated in Transmir v1.2. Nodes corresponding to miRNAs are in blue hexagons, nodes corresponding to TFs are in purple circles. Edges are represented as green arrows from TFs to target miRNAs.

**Figure 8.**
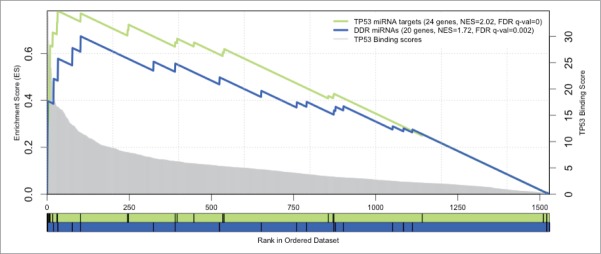
Enrichment plots of microRNA signatures in all miRNAs ranked by TP53 motifs. GSEA pre-ranked results for 2 gene sets (signatures) scored for enrichment of TP53 binding sites: the DDR miRNAs (in blue) and the TP53 annotated targets as a positive control set (in green). The X-axis represents all known miRNA genes ranked by the score of TP53 motifs in their regulatory region. Genes with best scores are toward the left. The score distribution is represented by the gray chart in the background, with corresponding y-axis in the right. The black vertical bars correspond to the ranking of the miRNAs of the given signature. The curves display the running enrichment score (ES) for the gene set as the analysis walks down the ranked list. The score at the peak of the plot (the score furthest from 0.0) defines the ES for the gene set. For each tested set, the number of miRNAs, the normalized enrichment score (NES) and the FDR q-value are indicated in the legend.

Next, we tested if the observed genome-wide changes in the expression of miRNA species after DNA damage were coordinated through one or more transcription factors (TFs). We first extracted 566 annotated and curated TF-miRNA interactions from the TransMir database,^28^ for all the DDR miRNAs, and found that p53 was the most connected TF, regulating 4 of the 12 connected DDR miRNAs (miR-34a, miR-34c, miR-215, miR-192) (**Fig. 7**). As the annotation of the TF-miRNAs interactions may not be representative of all the potential interactions, we proceeded to perform a more systematic analysis to evaluate the relevance of p53 in regulating the DDR miRNAs. We first used the *cisTarget* approach to score promoter sequences of all miRNAs with p53 position-weight matrices across 10 vertebrate genomes (Aerts et al., PLoS ONE 2007). Next, we tested whether the 20 most highly-induced DDR miRs are enriched at the top of this ranking using Gene Set Enrichment Analysis (GSEA)^29^ (see MATERIALS AND METHODS). The result is shown in **Fig. 8****.** We found that the upregulated miRNAs upon DDR are significantly enriched with a normalized enrichment score of 1.72 (nominal p-value < 0.001 and FDR q-value = 0.002) while our positive controls corresponding to known p53 miRNA target sets are found enriched with a normalized enrichment score of 2.02 (nominal p-value < 0.001 and FDR q-value < 0.001 for the curated p53 targets) and 1.76 (nominal p-value = 0.001 and FDR q-value = 0.002 for the annotated TP53 targets. The GSEA analysis of the 20 most highly-induced DDR miRNAs predicts 6 enriched miRNAs that contribute most to the enrichment score as direct p53 targets, including 3 annotated p53 targets (miR-125b-1 and miR-34a/c), as well as 3 novel p53 target miRNAs, namely miR-486, miR-139, and let-7a-2 **(****Table 2****)**. Interestingly, we also found the consistently repressed DDR miRNAs were highly enriched in this ranking (NES = 1.20, nominal p-value = 0.230 and FDR q-value = 0.273), essentially due to 3 well-ranked miRNAs that are potential p53 targets (miR-20a, miR-1273a and miR-374a). The repressed DDR miRNA set gives lower enrichment than the induced set, whereas the merged set gives an intermediate enrichment (NES = 1.64), suggesting that p53 may play a stronger role in the transcriptional activation of miRNAs after DNA damage rather than in their repression.

**Table 2.** Enriched miRNAs as transcriptional targets of p53

Name	Rank in gene list	Rank metric score	Runninges	Core enrichment
mir-34a	0	34.82690048	0.19905818	Yes
mir-34c	1	34.6026001	0.3968343	Yes
mir-486	19	18.5720005	0.49173445	Yes
mir-139	32	16.42160034	0.5776527	Yes
let-7a-2	76	12.94890022	0.623206	Yes
mir-125b-1	101	11.50020027	0.67305356	Yes
mir-365a	323	6.691860199	0.5650411	No
mir-215	389	6.109330177	0.556942	No
mir-96	524	5.189610004	0.497921	No
let-7a-1	651	4.54610014	0.44051638	No
mir-423	759	3.868499994	0.39181334	No
mir-1247	789	3.651760101	0.3934929	No
mir-192	872	3.154949903	0.35725677	No
mir-191	877	3.117209911	0.3724264	No
mir-3184	901	3.018699884	0.3744585	No
mir-152	1051	2.350739956	0.2892843	No
mir-1-2	1084	2.201610088	0.2806899	No
mir-148b	1111	2.113770008	0.27556428	No
mir-125b-2	1520	0.065534301	0.005919015	No
let-7a-3	1529	0.00653213	6.62E-04	No

**Figure 9.**
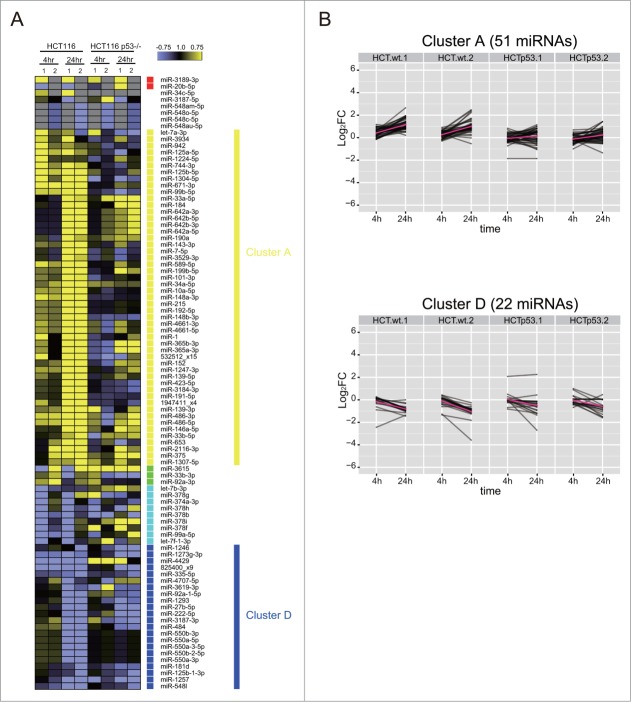
TP53 shapes genome-wide patterns of human miRNA expression during the DNA damage response. (**A**) Shows the heat map of expression patterns for 93 DDR-responsive miRNAs in HCT116 and HCT116*TP53-/-* cells at 4 h and 24 h after DNA damage, compared to their expression at 0 h. Two technical replicates (1 and 2) were analyzed for each condition. Each row represents a specific miRNA species. miRNAs were clustered by the pattern of the expression into clusters as in **Fig. 5**. (**B**) Shows changes in the expression of cluster A (miRNAs induced during the DDR) and cluster D (miRNAs repressed during the DDR). Note that the induction of cluster A miRNAs is lost in HCT116*TP53-/-* cells, but not the repression of those in cluster D.

These analyzes suggest that p53 plays an important role in shaping genome-wide changes in miRNA expression after DNA damage. We therefore used NGS and bio-informatics analyzes to document the scope of genome-wide changes in small, non-coding RNA expression in the cell line HCT116*TP53-/-*, an isogenic derivative of HCT116 in which *TP53* has been deleted by gene targeting.^30^ Western blotting confirms that HCT116*TP53-/-* cells still activate ATM Ser1981 phosphorylation after DNA damage, but no longer expresses the p53 protein (**Fig. 1B**). Moreover, qRT-PCR analyzes (**Fig. S3**) confirm that the expression of miRNAs belonging to the miR-34 group, which are known to be p53-dependent, is indeed significantly suppressed in HCT116*TP53-/-* cells when compared to HCT116 controls. To assess our hypothesis in detail, we investigated how p53 affects the patterns of DDR miRNAs, which were induced or repressed mainly at 24 h in HCT116, corresponding to the cluster A (51 miRNAs) or the cluster D (22 miRNAs), respectively (**Fig. 9**). More than 70% of the induced miRNAs in cluster A failed to be induced after DNA damage in HCT116*TP53-/-* cells, including the six induced miRNAs predicted as direct p53 targets. In cluster D, the repression was lost for 6 miRNAs at 4 h and for 5 members of the miR-550 family at 24 h. Among the seven miRNAs found robustly repressed in HCT116, 3 miRNAs were found to be p53 dependent (miR-1273a or 825400_x9, miR-374a and miR-4707-5p) including 2 previously predicted as direct p53 targets (**Fig. 9B**). These results suggest that miRNA expression during the DDR is both directly and indirectly p53-dependent, and identify several miRNAs as previously unrecognized targets for p53-dependent regulation.

## Discussion

The findings we report here provide a comprehensive description of genome-wide changes in the expression of miRNAs during the response to DNA breakage in human cells. Previous analyzes using alternative methods such as miRNA array profiling have detected a smaller fraction of DDR-regulated human miRNAs induced by ionising radiation. For instance, 11 DNA damage responsive miRNAs were detected in dermal fibroblasts, out of 361 tested,^31^ of which 2 (miR-20a, upregulated; miR-148b, downregulated) were also detected in our studies albeit with differing patterns of expression. Changes in the expression of 17 out of 186 miRNAs were detected in primary human fibroblasts exposed to ionizing radiation,^32^ of which only 2 (miR-125b and let-7a) were also found in our analysis. Analysis of several cancer cell lines detected 29 DDR-regulated miRNAs out of 1100,^33^ of which we detected 3 (miR-96, 148b and let-7a). Differences in the cell type and radiation dose, as well as the lower sensitivity of array-based methods compared to NGS, may account for these differences.

Our experiments were designed to detect DDR-induced changes in miRNA expression at 4 h and 24 h after damage exposure, in 3 different cell types. The choice of time points reflects published evidence that the activation of gene expression by transcription factors such as NFkB or p53 induced after DNA damage occurs within 4–24 h time-scale^1^; indeed, similar time points have also been used to study miRNA-mediated changes after UV-induced damage.^13^ The cell types were chosen to facilitate a series of biologically informative conclusions. Thus, MCF10A is a widely used, non-transformed mammary epithelial cell line, which retains normal G1 and G2 cell cycle checkpoints for DNA damage, and in which ATM activation and p53 induction occur after DNA damage (**Figs. 1A** and **B**). By contrast, HCT116 is a transformed colorectal carcinoma cell line, for which an otherwise isogenic but p53-negative counterpart has been generated by gene targeting (**Fig. 1B**), enabling the role of p53 in the response to be determined.

Our study design did not include time-point specific control samples that were not treated with IR; instead, we made comparisons with undamaged cells (0 h samples). Moreover, we have only performed technical replicates of library preparation and NGS from the same RNA samples, rather than to include multiple biological replicates at each time point. These procedures might increase the false positive rates.^34^ However, we have taken several steps to mitigate problems potentially arising from biological variance. We irradiated all samples together, and returned them to the incubator before preparing RNA samples at 4 h and 24 h to minimize differences in manipulation between samples to the extent possible in this study design. We sequenced, as noted above, replicate samples of miRNAs from several different cell lines at different times after exposure to IR. Finally, we used an independent method, qPCR, to assess changes in the expression of DDR-regulated miRNAs in biological replicate samples. Collectively, these steps provide an additional layer of controls to account for biological variance.

Our work demonstrates that complex and strongly cell-type dependent patterns of altered miRNA expression occur after the exposure of cells to DNA damage. This suggests that alterations in miRNA form an essential regulatory component during the DDR. For example, previous studies have shown that the miR-34 family is induced by DNA damage, and suppresses the expression of genes such as CDK4, CDK6 and cyclin E, regulating cell cycle arrest after DNA damage.^7^ Notably, many miRNAs detected in our work are induced >4–24 h after damage, suggesting that their turnover may coordinate essential changes in gene expression required for the DDR over a relatively long time period.^35^ Thus, our findings provide a genomic resource for future biological studies to uncover new participants and mechanisms in the human DDR, which operate not only during the acute phase of the response, but also during cell recovery.

However, the strongly cell-type dependent patterns of miRNA expression detected in our work emphasizes an important issue that such future studies must be mindful of. We observe that hardly any miRNAs are commonly regulated in the different cell lines used here, even in the 2 *TP53* wild-type epithelial cell lines MCF-10A and HCT116. Given that different cell types derived from different tissues show clear differences in sensitivity to IR and the dynamics of the DDR, likely through differential use of DDR signaling and DNA repair pathways,^36^ our findings caution that each cell type may regulate an individual subset of miRNAs during the DDR.^37^ This could make it difficult in future studies to compare or extrapolate the physiological role of DNA damage-responsive miRNAs between different cell types.

Several miRNAs detected in our analysis as being induced after DNA damage exhibit lower expression in different forms of epithelial cancer. For example, miR-125b expression is decreased in breast cancer,^38^ miR-148b, in gastric cancer^39^; miR-148a and miR-30a-5p, in colorectal cancer^40,41^; and miR-96, miR-143/145, and miR-34a, in pancreatic cancers.^42,43,44^ This correlation prompts us to speculate that these miRNAs may act as tumor suppressors, whose induction is normally part of the DDR, but is lost during carcinogenesis to induce genome instability. Further studies to address this hypothesis are warranted.

We were able to analyze if the DDR miRNA expression levels in tumor samples predict clinical outcomes in patients using MIRUMIR, a webserver that performs survival analyzes across several available data sets.^45^ Around half of the DDR miRNAs exhibited a significant association between their expression levels and patient survival (Chi-squared corrected p-value < 0.05) in cancers of the prostate, breast and nasopharynx. These include 24 miRNAs out of the 51 DDR-induced miRNAs in HCT116 (Cluster A in **Fig. 5**), 1 out of 4 DDR miRNAs from Cluster B, 2 out of 4 miRNAs from Cluster C, and 6 out of 10 DDR miRNAs from Cluster D (Supplementary Table S2). Also, 2 out of 4 predicted direct targets of p53 - miR-34a and miR-215 — are also significantly associated with patient survival (**Fig. S4**). The majority of these associations (37/50) linked low expression of the DDR miRNAs with poor clinical outcome. However, in few cases (let-7a*, miR-33b, miR-34a, mir-143, miR-423, miR-486, miR-92a, miR-484), a high level of miRNA expression was associated with poor outcome, but in a different cancer type. For example, low expression levels of miR-34a, one of the predicted direct targets of p53, are associated with poor outcome in breast cancer (p-value = 0.0009), while high expression levels are associated with a poor outcome in ovarian carcinoma and hepatocellular carcinoma (p-values = 0.049) (**Fig. S4**). These results emphasize that tissues-specific patterns of DDR-induced miRNA expression may provide prognostic markers for patients with cancer.

Our findings reveal a major biological function for the p53 tumor suppressor in regulating the induction of miRNA expression following DNA damage. In particular, we find that 51 DDR-miRNAs (represented by cluster A in **Fig. 9B**) were induced at 24 h in HCT116 cells but not in their *TP53-/-* but otherwise isogenic counterparts. It has been proposed that p53 is involved in miRNA biogenesis in conjunction with the Drosha complex.^14^ In addition, our analyzes suggest that p53 acts in the DDR via a transcriptional mechanism, exemplified in the enrichment of p53 consensus binding sites in the regulatory regions of several DDR miRNAs. This function of p53, along with the possible roles played by other DDR-induced transcription factors, deserves attention in future studies. For example, the recent availability of ‘big’ cancer data sets such as TCGA will make it possible to interrogate at what level p53 status (mutations, expression, subtypes) is correlated to its miRNA direct targets, and to patient survival.

Thus, in conclusion, the work we report here provides a first outline of genome-wide changes in the expression of miRNAs during the response of human cells to DNA damage, and reveals an important biological role for the tumor suppressor p53. We demonstrate that >10% of all known miRNAs alter their expression 4–24 h after exposure to radiation, in complex patterns that strongly depend on cell type. Binding sites for p53 are enriched in the regulatory regions of the most-highly induced miRNAs, and genetic ablation of *TP53* decreases miRNA induction but not suppression, during the DDR. The expression levels of certain DDR-induced miRNAs in tumor samples correlate with patient outcome. Our findings provide a genomic resource for future work studying the biological significance of miRNAs that are induced or repressed after exposure to DNA damage, identifying their targets and the role of their regulation, and elucidating the function of p53 in the transcription of DDR-induced miRNAs.

## Materials and Methods

### Cell culture

MCF10A, a widely used, non-transformed mammary epithelial cell line, which retains normal G1 and G2 cell cycle checkpoints for DNA damage, and in which ATM activation and p53 induction occur after DNA damage, was obtained from, and authenticated by, Cancer Research UK (CRUK) Cell Services. HCT116 is a transformed colorectal carcinoma cell line, for which an otherwise isogenic p53-negative counterpart has been generated by gene targeting, enabling the role of p53 in the response to be determined. HCT116 and HCT116 *TP53*-/- cell lines were kindly provided by Dr. Bert Vogelstein (Sidney Kimmel Comprehensive Cancer Center, Baltimore, MD, USA). MCF10A cells were grown in Dulbecco’s Modified Eagle’s medium (DMEM/F-12 (1:1), Invitrogen) supplemented with 5% horse serum, 10 mg/ml insulin, 20 ng/ml EGF, 100 ng/ml choleratoxin, 500 ng/ml hydrocortisone and 100 U/ml penicillin and streptomycin. HCT116 and HCT116 *TP53*-/- cells were cultured in McCoy’s 5A Medium (Invitrogen) supplemented with 10% FBS, 100 U/ml penicillin and streptomycin. Cultures were maintained at 37°C and at 5% CO_2_.

### DNA damage treatment

The day before IR treatment, 1.2 × 10^6^ MCF10A cells or 1.8 × 10^6^ HCT116 and HCT116 *TP53*-/- cells were plated in 9.2 cm dishes. Five Gy of IR was administered using the 43855D Faxitron Cabinet X-ray System, followed by incubation for 4 or 24 hour at 37°C in 5% CO^2^ before harvesting.

### Western blotting

Cells were harvested with trypsin and collected by centrifugation in the presence of growth media before washing in phosphate buffer saline (PBS) washes. Whole-cell extracts were made in the NP-40 lysis buffer [50 mmol/L HEPES (pH = 7.4), 100 mmol/L NaCl, 0.5% NP-40, 10 mmol/L EDTA, 20 mmol/L β-glycerophosphate, 1 mmol/L DTT, 1 mmol/L sodium orthovanadate, 1 mmol/L PMSF (phenylmethylsulfonylfluoride), complete protease inhibitor cocktail (Roche, Burgess Hill, UK)]. Samples were resolved with NuPAGE Novex 4–12% Bis-Tris gels (Invitrogen, Paisley, UK). Western blot detection with the listed antibodies was visualized with horseradish peroxidase (HRP)-coupled secondary antibody and an enhanced chemiluminescence kit (Amersham, Bucks, UK). Antibodies used were: phospho-ATM (Ser1981) (D6H9) Rabbit mAb (Cell signaling #5883) at 1:500; anti-β-actin (A5441, Sigma) at 1:5000; anti-p53 (sc-126, DO-1, Santa Cruz, CA, USA) at 1:1000; phospho-p53 (Ser15) (16G8) Mouse mAb (Cell signaling #9286) at 1:500.

### Total RNA extraction

Cells were rinsed once with 1xPBS, and lysed in 3 ml of Trizol reagent (Invitrogen, Carlsbad, CA, USA). RNA extraction was carried out according to the manufacturer’s instructions. The concentration of purified total RNAs was adjusted to 1 □g/□l with RNase free water. RNA was extracted once from each of the 3 cell lines (MCF10A, HCT116 and HCT116 p53-/-) at 0 h, 4 h or 24 h after DNA damage. Small RNA (18–30 nt) purified from these samples by gel electrophoresis was independently analyzed twice using RNA sequencing. All analyzes using qPCR for miRNA expression were performed using at least 3 independent biological replicates without small RNA fractionation.

### Generation of the small RNA library for NGS

Ten μg of total RNA was subjected to 15% TBE-urea PAGE before small RNA species between 18–30 nt in length were excised and eluted in 0.3 M NaCl. The v1.5 small RNA 3′ adapter (Illumina) was ligated using T4 RNA ligase 2, truncated (NEB), and then the small RNA 5′ adapter was ligated using T4 RNA ligase. Reverse transcription was performed using the small RNA RT primer (Illumina) using the 5′ and 3′ adapter-ligated RNAs as a template. Twelve cycles of PCR amplification were carried out using GX1 and GX2 primers with Phusion DNA polymerase. The amplified cDNA preparation was subjected to 6% TBE PAGE, and species of ∼100 bp were excised and purified. Finally, each cDNA library was validated with an Agilent bioanalyzer using the DNA 1000 kits to confirm size, purity, and concentrations. Each library was sequenced in 2 lanes on the Illumina Genome Analyzer IIx using a single-read and 36 sequencing cycles. Raw data are available from the NCBI Gene Expression Omnibus (GEO) repository (accession number GSE50064) and the reviewers can access the data through this reviewer access link http://www.ncbi.nlm.nih.gov/geo/query/acc.cgi?token=fjsvtqsieeqiahi&acc=GSE50064.

### Quantitative reverse transcription (qRT)-PCR

The total RNA (10 µg) was treated with DNase I (Promega) followed by polyadenylated using Poly(A) polymerase (NewEngland BioLab) according to the manufacturer’s instructions, phenol:chloroform extracted, ethanol-precipitated, and dissolved in RNase-free water at 1 μg/μl. A modified cDNA was made as follows: 10 µg of polyadenylated RNA was reverse-transcribed using Superscript II reverse transcriptase (Invitrogen) with 2.5 µg of random hexamers (Qiagen) and 500 ng of oligo(dT) adapter primer (5′-GCGAGCACAG AATTAATACGACTCACTATAGGTTTTTTTTTTTTVN-3′). The reaction was terminated by incubation at 70°C for 10 min and diluted into 2 mL of dH_2_O (5 µg/mL). Quantitative PCR was used to measure the mature miRNA as follows: 10 µL of 2 × LightCycler 480 SYBR Green I Master (Roche) was mixed with 5 p.m.ol of both the forward and reverse primers in a volume of 15 µL and 5 µL of cDNA was added in a final volume of 20 μL. Basically, each forward primer has each microRNA specific sequences and the reverse primer was universal for all the cDNA from microRNAs and the sequence was Uni-R, 5′-GCGAGCACAGAATTAATACGACTCAC-3′. All reactions were run in triplicate on LightCycler 480 (Roche) using a PCR condition for 5 min at 95°C, 10 sec at 95°C 10 sec at 56°C, 10 sec at 72°C, for 45 cycles. A reference gene, SNORD48 small nucleolar RNA, C/D box 48 (RNU48-F, 5′-GTGTCGCTGATGCCATCAC-3′; RNU48-R, as same as the universal reverse primer) was used to confirm the similar amount of small RNA in different samples. Expression levels were calculated using the “Second Derivative Maximum Method” according to software provided for the Light Cycler 480 (Roche) to quantitate DDR miRNAs or the control RNU48.

### Detection of known miRNA species

Nine libraries of ∼12 million reads each were generated in duplicate from next-generation sequencing of small RNA samples. Adapters were trimmed from sequencing reads. Reads of length <18 nt after adapter trimming and with low complexity (polyN) were excluded from further analyzes. The remaining reads were mapped to the human genome (Hg18) using Bowtie^46^ with the default parameters (seed length of 28, maximum number of 2 mismatches, and report up to 1 alignment per read). Reads mapping the reference genome were then aligned to known miRNAs (miRBase version 18, release 11/2011^47^) using BLAST,^48^ where 100% identity between reads and known miRNAs sequences was required. To detect other known small RNAs, the mapped reads were also aligned with BLAST against the non-coding RNAs extracted from the UCSC Table Browser [PMID:11081512,PMID:11914277]. A weight of 1 was added to miRNA when no reads was detected in a given sample.miRNAs with an aggregate count of less than 100 in all 16 samples were eliminated; then the total read count for each lane was scaled relative to the library size (total number of reads that mapped to known miRNAs). Read counts of technical replicates were then merged, and log2 (fold change) values were calculated for each miRNA. P-values were subsequently calculated using a binomial approximation to Fisher’s exact test for each miRNA.

### New microRNA predictions

New miRNA were predicted using MIReNA version 2.0, a genome-wide search algorithm designed for the discovery of new microRNAs from deep sequencing data (Mathelier and Carbone 2010). The algorithm was used with default parameters (using option–-blast to perform the blast search of deep sequencing reads on the Human genome Hg18, and option–-miRNAs with the conserved mature miRNAs sequences). All filtered reads were merged in one input file and reads with only one sequencing count were discarded for this step.

### Differential expression analysis

Counts for known miRNAs were calculated as the sum of the reads matching to the miRNA. 308 Predicted miRNAs were added. A threshold on the aggregate count (100) was used to discard low expressed miRNAs over the 18 sequenced samples, leaving 965 miRNAs (including 125 new miRNAs). Then, the total read count for each lane was scaled relative to the library size. The significance of the differential expression was calculated for each miRNA using the Fisher test function from R stats package. P-values were corrected for multi-testing (total number of miRNA tested). We used less stringent thresholds to filter the DDR new miRNAs: |log2 (fold change)| >0.5, log2 (average read counts) >4, and significance (p < 0.001) in at least one out of 4 conditions (MCF10A 4 and 24 hours, HCT116 4 and 24 hours, compared to their respective control). All data analysis was done using R (R Development Core Team, 2011).

### Filtering for the most-highly regulated DDR miRNAs

The initial list of DDR miRNAs corresponded to known and new miRNAs, which show significant differential expression at least once over all comparisons and different replicates. This led us to a list of 150 miRNAs. To get the most robust predictions we applied additional filters: 1) the fold change of the miRNAs should show a similar trend at 4 h and 24 h, 2) the fold change at 4 h or 24 h should be significant (multi-testing corrected p value < 0.05), and 3) miRNAs should be significantly induced in 2 cell lines or/and in 2 replicates. This filtering detected 23 induced DDR miRNAs (including one new miRNA). Three of the DDR miRNAs were discarded as they correspond to different species of the same miRNA. We kept the mature sequences that may represent the functional species (miR-139-5p, miR-486-5p) instead of the star sequences (miR-139-3p, miR-486-3p). The mature sequences of miR-365a-3p and miR-365b-3p are exactly the same, while our differential expression analysis does not take the mapping position of the reads into account and thus does not allow us to distinguish the right species. Note that we kept the mature sequences of miR-148a-3p and miR-148b-3p as they differ from 2 nucleotides, idem for miR-34a-5p and miR-34c-5p (4 mismatches, 1 insert).

### p53 motif enrichment

We used known p53 motifs to detect homotypic *cis*-regulatory modules (CRMs) in the putative regulatory regions of human miRNAs using the Cluster-Buster v1.5 CRM-scanning tool [1]. We delineated miRNA promoter regions to cover the genomic regions [−5 kb,+500 bp] in relation to the start of single or clustered intergenic miRNAs, [−4 kb,+2 kb] to the transcription start sites of host genes for intragenic miRNAs, and [−3.5 kb,−500 bp] to the start of single or clustered intragenic miRNAs. We defined a miRNA as being intragenic if it was located within an annotated transcript from the same strand (host gene). From 1523 annotated miRNAs in miRBase v18 [2], we extracted 383 intergenic features (30 clusters and 353 single miRNAs) and 2766 intragenic features (388 clusters and 2378 single miRNAs). Seven annotated Position Weight Matrices (PWMs) corresponding to p53 binding were collected from Jaspar core database^49^ and TRANSFAC Professional Release,^50^ [license number] (transfac_pro-M01655, transfac_pro-M01652, transfac_pro-M00272, transfac_pro-M01651, transfac_pro-M00761, transfac_pro-M00034 and jaspar-MA0106.1). The miRNA regulatory regions were scored for p53 homotypic CRMs using cluster-buster (using –c0, setting the cluster threshold to zero, in order to score all regions). The maximum score for each miRNA was used to rank them in a given species. To benefit from the conservation of regulation, putative orthologous regulatory regions in 10 related species were deduced using the UCSC LiftOver standalone program, as described before for cisTarget.^51^ In total, we scored the miRNAs in 10 vertebrate species: *Bos taurus* (bosTau4), *Canis familiaris* (canFam2), *Homo sapiens* (hg19), *Mus musculus* (mm9), *Monodelphis domestica* (monDom5), *Pan troglodytes* (panTro2), *Pongo abelii* (ponAbe2), *Macaca mulatta* (rheMac2), *Danio rerio* (rn4), *Sus scrofa* (susScr2). The 10 rankings were integrated into one final q-value score using rank aggregation by order statistics,^51,52^ followed by a minus log transformation producing a final ranking of all Human miRNAs. We then applied Gene Set Enrichment Analysis (GSEA)^29^ on the 3 sets of miRNAs: 20 DDR predicted miRNAs, 13 TP53 target miRNAs annotated in Transmir (Transmir set), and 24 curated TP53 target miRNAs (mean div normalization, default weighted scoring scheme as parameters). Annotated TF-miRNAs interactions are from TransmiR v1.2 (162 miRNAs, 37 TFs). The curated set corresponds to TP53 sets annotated in Transmir (http://202.38.126.151/hmdd/mirna/tf/), HMDD (http://202.38.126.151/hmdd/mirna/md/) and from ref (http://www.ncbi.nlm.nih.gov/pubmed/22110125).

### Survival analysis from miRNA expression profiles

MIRUMIR (http://www.bioprofiling.de/GEO/MIRUMIR/mirumir.html) was queried online to test whether each DDR microRNA could predict survival across multiple available data sets covering 7 cancer types: breast cancer (GSE37405, GSE22216, GSE19783), prostate cancer (GSE21036), nasopharyngeal carcinoma (GSE36682), ovarian carcinoma (GSE27290), hepatocellular carcinoma (GSE10694), esophageal adenocarcinoma or squamous cell carcinoma (GSE13937) and lung cancer (GSE16025). For each available data set, samples were split into 2 groups, with high or low microRNA expression. MIRUMIR provides Kaplan-Meier (KM) plots along with Cox regression survival analyzes.

## Authors’ Contributions

HH and ARV designed the study. HH and RJ performed the experimental work. HH, RJ and ARV analyzed the data, and wrote the paper. WN made logistical arrangements for the next-generation sequencing of 6 samples. SA and MMB assisted in the data analysis. SA provided comments on the manuscript.
