# Supplementary material for: p53 shapes genome-wide and cell type-specific changes in microRNA expression during the human DNA damage response
Source: Cell Cycle. 2014 Oct 30;13(16):2572–86. doi: 10.4161/15384101.2015.942209 (PMC4601526; doi:10.4161/15384101.2015.942209)
Supplement: KCCY_942209_supplemental_file.pdf [file kccy-13-16-942209-s001.pdf]

SUPPLEMENTARY MATERIAL

p53 shapes genome-wide and cell type-specific changes in microRNA expression during the human DNA damage response

Hiroyoshi Hattori, Rekin's Janky, Wilfried Nietfeld, Stein Aerts, M. Madan Babu, and Ashok R. Venkitaraman

Supplementary Tables 1 and 2

Supplementary Figure legends

Supplementary Figures S1-S4

Supplementary Table 1, related to Figure 6. <u>miRNA clustering and DDR-induced changes in expression.</u> 'DDR' signifies those miRNAs whose expression is significantly altered at 4 or 24 h after DNA damage using the statistical criteria noted in Methods, and 'non-DDR', miRNAs that do not exhibit such alterations.

	DDR	non-DDR	total
clustered miRNAs	53	92	145
single miRNAs	264	1122	1386
total	317	1214	1531

Supplementary Table 2, related to Figure 5. Correlations between DDR miRNA expression and patient outcome in different cancer types.

Cluster	MicroRNA	Type of Cancer	Expression in poor outcome*	P value **	GEO ID	Array ID
A	HSA-LET-7A*	prostate cancer	High	0.000099	GSE21036	HSA-LET-7A*
	HSA-LET-7A*	breast cancer	Low	0.02295	GSE37405	17888
	HSA-MIR-1	prostate cancer	Low	0.0000181	GSE21036	HSA-MIR-1
	HSA-MIR-1	breast cancer	Low	0.0000505	GSE37405	42573
	HSA-MIR-33A	breast cancer	Low	0.00058	GSE37405	42716
	HSA-MIR-33A	breast cancer	High	0.0056	GSE22216	ILMN_3167691
	HSA-MIR-33B	prostate cancer	High	0.000002472	GSE21036	HSA-MIR-33B
	HSA-MIR-33B	breast cancer	High	0.02439	GSE22216	ILMN_3166988
	HSA-MIR-33B	breast cancer	Low	0.047	GSE37405	42715
	HSA-MIR-34A	breast cancer	Low	0.000899	GSE37405	27217
	HSA-MIR-34A	ovarian carcinoma	High	0.049	GSE27290	181
	HSA-MIR-34A	hepatocellular carcinoma	High	0.049	GSE10694	135
	HSA-MIR-99B	breast cancer	Low	0.0007208	GSE37405	11184
	HSA-MIR-125A-5P	breast cancer	Low	0.02208	GSE37405	10928
	HSA-MIR-139-3P	breast cancer	Low	0.00378	GSE37405	42451
	HSA-MIR-143	prostate cancer	Low	0.0005368	GSE21036	HSA-MIR-143
	HSA-MIR-143	hepatocellular carcinoma	High	0.00108	GSE10694	187
	HSA-MIR-143	breast cancer	Low	0.013226667	GSE37405	13177
	HSA-MIR-148B	breast cancer	Low	0.001024	GSE37405	19585
	HSA-MIR-184	breast cancer	Low	0.0002758	GSE37405	10978
	HSA-MIR-190	breast cancer	Low	0.04116	GSE37405	27536
	HSA-MIR-191	lung cancer	High	0.03416	GSE16025	BM10968
	HSA-MIR-199B-5P	prostate cancer	Low	0.00000912	GSE21036	HSA-MIR-199B-5P
	HSA-MIR-199B-5P	breast cancer	Low	0.0384	GSE37405	19591
	HSA-MIR-215	breast cancer	Low	0.0003924	GSE37405	11015
	HSA-MIR-365	breast cancer	Low	0.0001	GSE37405	11078
	HSA-MIR-375	breast cancer	Low	0.02504	GSE37405	42498
	HSA-MIR-423-5P	breast cancer	Low	0.000051	GSE37405	27565
	HSA-MIR-423-5P	prostate cancer	High	0.006075	GSE21036	HSA-MIR-423-5P
	HSA-MIR-486-5P	breast cancer	Low	0.00972	GSE37405	32946
	HSA-MIR-486-5P	prostate cancer	High	0.0142	GSE21036	HSA-MIR-486-5P
	HSA-MIR-642	breast cancer	Low	0.000508	GSE37405	42679
	HSA-MIR-642	breast cancer	Low	0.00382	GSE22216	ILMN_3168055
	HSA-MIR-671-3P	breast cancer	Low	0.00975	GSE37405	42525
	HSA-MIR-942	breast cancer	Low	0.0145	GSE37405	42608
	HSA-MIR-1224-5P	nasopharyngeal carcinoma	Low	0.01018	GSE36682	946
	HSA-MIR-1304	nasopharyngeal carcinoma	Low	0.00192	GSE36682	458
B	HSA-MIR-92A	prostate cancer	High	0.01002	GSE21036	HSA-MIR-92A
	HSA-MIR-92A	breast cancer	Low	0.0342	GSE22216	ILMN_3166986
C	HSA-LET-7B*	breast cancer	Low	0.01005	GSE37405	42769
	HSA-MIR-99A	breast cancer	Low	0.034	GSE22216	ILMN_3168213
D	HSA-MIR-125B-1*	nasopharyngeal carcinoma	Low	0.0429	GSE36682	16
	HSA-MIR-125B-1*	breast cancer	Low	0.000442	GSE37405	17876
	HSA-MIR-335	nasopharyngeal carcinoma	High	0.046	GSE36682	68
	HSA-MIR-484	breast cancer	Low	0.00029715	GSE37405	42698
	HSA-MIR-484	prostate cancer	High	0.00004109	GSE21036	HSA-MIR-484
	HSA-MIR-550	nasopharyngeal carcinoma	Low	0.01494	GSE36682	370
	HSA-MIR-550	breast cancer	Low	0.00000048	GSE37405	42914
	HSA-MIR-1257	nasopharyngeal carcinoma	Low	0.0114	GSE36682	953
	HSA-MIR-1293	nasopharyngeal carcinoma	Low	0.0329	GSE36682	711

*Expression levels in tumour specimen from patients with poor outcome.

**All the listed miRNAs have $p < 0.05$ with the Chi square test. Corrected p values by false discovery rate (FDR) are given.

Supplementary Figure Legends

Supplementary Figure 1, related to Figure 1. Data analysis pipeline and outline results. **(A)** Flowchart of the data analysis pipeline for the discovery of DDR-regulated miRNAs**.** Blue boxes represent the steps in the data analysis pipeline. White boxes represent the inputs and outputs. **(B)** The up-regulated miRNAs after IR are categorised into 4 conditions; MCF10A two replicates, MCF10A-1 (red) and MCF10A-2 (blue), HCT116 two replicates, HCT116-1 (orange) and HCT116-2 (green) using a Venn-diagram. Twenty-three miRNAs (numbers in black) belong to at least 2 conditions are selected as the most robustly induced miRNAs. **(C)** The down-regulated miRNAs after IR are categorised into 4 conditions with a Venn diagram similar manor with **B**. The ten miRNAs satisfy 2 conditions as the robustly decreased miRNA after DNA damage.

Supplementary Figure 2, related to Figure 2. Analysis of DDR miRNAs by qRT-PCR and concordance with next-generation sequencing.

The $\log_2$ values (M values) of the fold changes at 4 and 24hour after DNA damage, which were measured by the next generation sequencing (NGS) and Q-RT-PCR (qRT-PCR) in 20 miRNAs with the two cell lines are colour coded in each cell. The concordance between the sequencing and qRT-PCR are determined if it is true (T) or failed (F) individually by the cell lines.

NA represents the data are not applicable.

Supplementary Figure 3, related to Figure 9. qRT-PCR analysis of TP53-regulated miRNAs.

miR-34 group miRNA, miR-34a and miR-34c are measured in HCT116 and HCT116*TP53-/-* cells at 0, 4, and 24hour time points. For the normalization of the mount of each template, the cDNA which synthesised from the same amount of total RNA between the targets are used. In addition, RNU48, a small nucleolar RNA, is offered as an internal control to confirm the equal amount of total RNAs are applied in each qRT-PCR reaction. Y-axis depicts the relative miRNA

levels, which normalized to 0 hour time point in each cell line and target miRNA. The error bar represents standard deviation (S.D.) from the experiments in triplicate.

Supplementary Figure 4, related to Figure 7. Correlations between patient survival and the expression levels of DDR miRNAs under TP53 regulation in tumour samples.

TP53 transcription factor networks of the DDR miRNAs were selected from the analysis shown in Figure 7. Four miRNAs (miR-34a, -34c, -192 amd -215) are regulated by the TP53 transcription factor; of these, miR-34a and miR-215 showed a correlation with patient survival. The Kaplan-Meier survival analysis was performed in cancer patients according to the expression levels of miR-34a (**A**, **B**, **C**) or miR-215 (**D**) in tumour samples. The survival curve in green represents the patients with lower expression of DDR miRNAs, whereas the curve in red shows those with higher expression. The X-axis represents the duration of survival after diagnosis. The Y-axis shows the probability of patient survival. **A**, Breast cancer, p value (corrected by FDR)=0.000899 (GEO dataset ID: GSE37405). **B**, Ovarian carcinoma, p value (corrected by FDR)=0.049 (GEO dataset ID: GSE27290). **C**, Hepatocellular carcinoma, p value (corrected by FDR)=0.049 (GEO dataset ID: GSE10694). **D**, Breast cancer, p value (corrected by FDR)= 0.0003924 (GEO dataset ID: GSE37405).

A

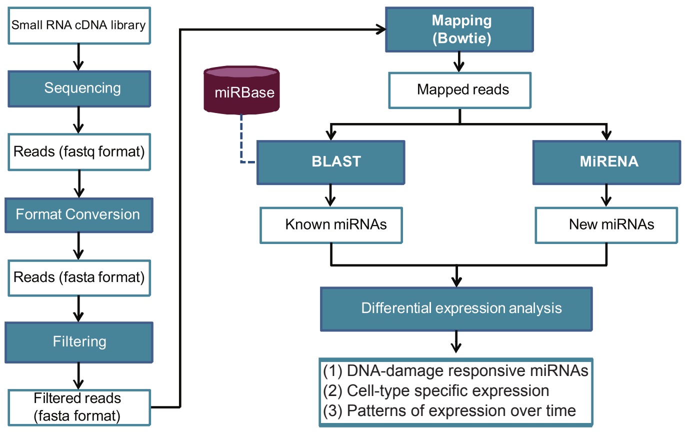

B

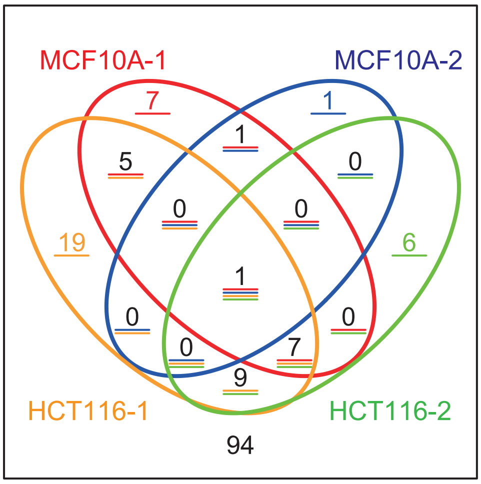

C

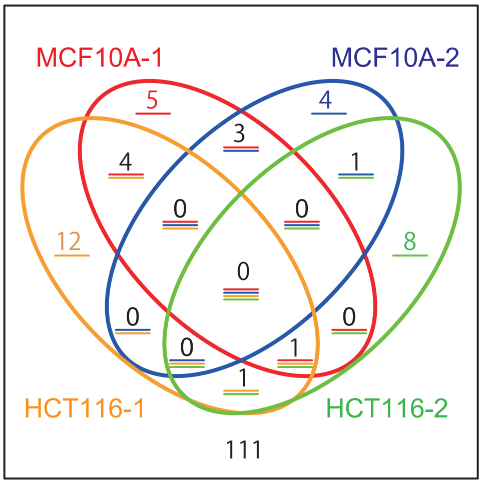

Supplementary Figure 1. Hattori et al

	NGS				qRT-PCR				Concordance	
	MCF10A		HCT116		MCF10A		HCT116		MCF10A	HCT116
miRNAs	4hr	24hr	4hr	24hr	4hr	24hr	4hr	24hr		
miR-34c-5p	2.37	3.98	0.59	3.54	0.81	1.93	0.39	1.42	*T*	*T*
1947411_x4	0.374	0.953	0.589	2.652	NA	NA	NA	NA	NA	NA
miR-486-5p	Inf	Inf	0.58	1.95	0.13	0.19	0.00	0.00	*F*	*F*
miR-148a-3p	1.10	1.77	0.71	1.26	0.21	1.02	-0.01	0.22	*T*	*F*
miR-152	0.33	0.85	0.23	1.76	0.56	1.59	-0.06	0.59	*T*	*T*
miR-1247-3p	NA	NA	0.525	1.69	NA	NA	NA	NA	NA	NA
miR-1	1.23	0.98	0.77	1.58	-0.05	0.66	0.00	0.59	*T*	*T*
let-7a-3p	1.554	0.489	0.89	0.496	NA	NA	NA	NA	NA	NA
miR-365a-3p	0.91	0.16	0.47	1.43	0.41	1.26	0.06	0.65	*T*	*T*
miR-3184-3p	1.102	0.297	0.214	1.392	NA	NA	NA	NA	NA	NA
miR-423-5p	1.10	0.30	0.21	1.39	0.37	0.83	0.11	0.02	*T*	*F*
miR-139-5p	1.00	-1.00	0.40	1.23	0.21	0.01	0.11	0.24	*F*	*F*
miR-125b-5p	-0.54	0.89	1.02	0.87	0.24	0.95	-0.01	0.38	*F*	*F*
miR-191-5p	0.04	0.29	0.17	0.97	0.01	0.45	0.01	0.65	*T*	*T*
miR-34a-5p	0.47	0.77	0.32	0.97	0.45	1.42	0.34	1.44	*T*	*T*
miR-96-5p	0.96	0.93	0.51	0.40	0.44	1.01	0.28	0.46	*T*	*T*
miR-192-5p	0.18	0.44	0.40	0.93	0.07	0.64	-0.14	0.47	*F*	*F*
miR-215	0.19	0.46	0.40	0.93	-0.11	0.43	-0.25	0.35	*F*	*F*
miR-148b-3p	0.28	0.26	0.43	0.84	0.50	1.22	0.05	0.45	*F*	*F*
miR-146a-5p	-0.644	-0.6	0.158	0.839	NA	NA	NA	NA	NA	NA

Supplementary Figure S2. Hattori et al

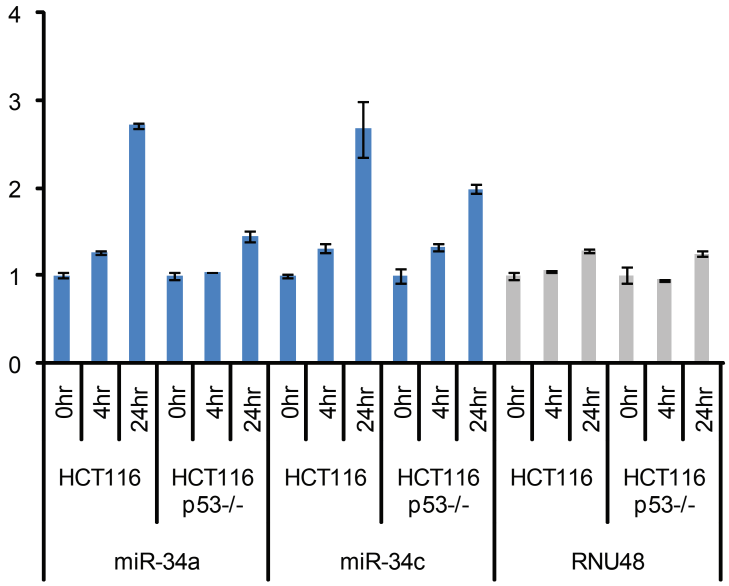

Supplementary Figure S3. Hattori et al

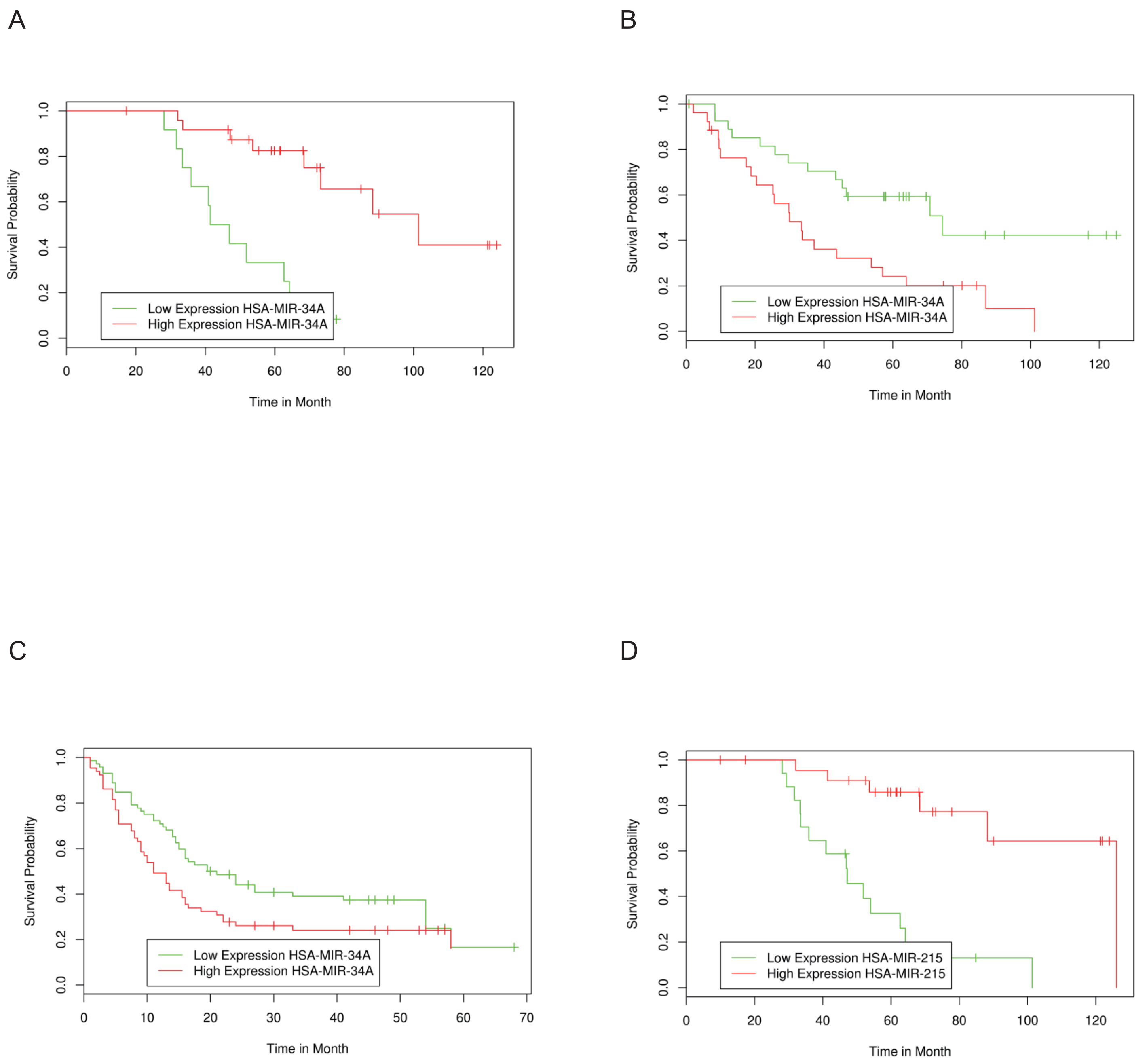

Supplementary Figure S4. Hattori et al
